# Perceptions, Experiences, and Beliefs About Patient Portals Among Women With Limited English Proficiency: Multicultural Qualitative Interview Study

**DOI:** 10.2196/60699

**Published:** 2025-02-26

**Authors:** Hae-Ra Han, Joyline Chepkorir, Tina Kim, Martha Zamora, Emma Huang

**Affiliations:** 1 Johns Hopkins University School of Nursing Baltimore, MD United States; 2 Johns Hopkins University Bloomberg School of Public Health Baltimore, MD United States

**Keywords:** patient portal, limited English proficiency, immigrant, women, mobile phone

## Abstract

**Background:**

Individuals in the United States with limited English proficiency (LEP) experience a disproportionate disease burden. Patient portals provide patient education, outreach, and linkage to preventive health services. While patient portals have been found to be effective in facilitating the use of preventive services, they have predominantly engaged well-educated, English-speaking, White populations. There is limited research investigating experiences and beliefs about patient portals among populations with LEP.

**Objective:**

This study aims to explore perceptions, experiences, and beliefs about patient portals among women with LEP.

**Methods:**

We used a qualitative semistructured interview design and recruited a purposive sample of women through diverse methods. The interview guide covered topics including experiences with patient portals, the perceived feasibility and relevance of patient portals, and perceptions of patient portals targeted toward women with LEP for health promotion. The interviews were audio recorded for verbatim transcription and analysis. Each bilingual interviewer reached data saturation after interviewing 12 (43%), 9 (32%), and 7 (25%) Korean-, Spanish-, and Swahili-speaking women, respectively, yielding a total of 28 women in the study*.*

**Results:**

We identified 4 main themes that were common across all linguistic groups: perceived benefits of patient portals, perceived facilitators of patient portal use, perceived barriers to patient portal use, and preferred features and suggested improvements. *Perceived benefits of patient portals* had 5 subthemes: easier communication with health care providers and health systems, getting connected and staying connected with health systems, easier and efficient access to one’s health records over time, staying informed of and engaged with one’s health and health management, and better patient engagement in medical visits. Subthemes for *perceived facilitators of patient portal use* were availability of time, widespread use and availability of smartphones and the internet in the United States, family support, and parenthood. Subthemes for *perceived barriers to patient portal use* were limited digital literacy and limited access to technology, LEP, lack of awareness and knowledge about patient portals, and illiteracy. Finally, subthemes for *preferred features and suggested improvements* were expanded language access to accommodate non-English speakers, improved accessibility to health information using graphics and patient education materials, and user onboarding education and technical support. Of note, while most subthemes were shared across all 3 groups, the widespread use and availability of smartphones and the internet in the United States and illiteracy subthemes were unique to Swahili-speaking women.

**Conclusions:**

Women with LEP recognized multiple benefits of patient portals; however, several barriers were also identified. These included limited digital literacy, restricted access to technology, LEP, and illiteracy. Barriers to patient portal use were closely tied to social determinants of health, which are commonly experienced by women with LEP. To expedite the attainment of health equity, it is important to promote access to health resources such as patient portals.

## Introduction

### Background

Racial and ethnic minority individuals in the United States, particularly those with limited English proficiency (LEP) [1], experience significant health disparities; for example, despite high incidence rates of cervical cancer, early detection through regular screening remains low among these populations, especially among women with LEP [2-4]. As a result, racial and ethnic minority women often present with more advanced disease stages than non-Hispanic White women [5]. This is largely due to language and cultural barriers, as well as low health literacy, which hinder their ability to navigate the US health system effectively [1].

One of the innovations in health care is the patient portal. Patient portals offer numerous benefits, including access to diagnosis, treatment information, and other health care resources, which empower patients by enhancing their knowledge and autonomy; for example, a recent systematic review of 10 studies on asynchronous patient–health care provider communication through patient portals found that electronic messaging with health care providers was associated with clinical benefits for patients with breast cancer, including improved screening behaviors [6]. Similarly, another systematic review of 24 patient portal interventions revealed that patient portals are generally effective in improving psychological outcomes (eg, health knowledge, self-efficacy, and decision-making), medication adherence, and the use of preventive services [7]. However, the review also noted that most patient portal interventions have predominantly engaged highly educated, English-speaking, White populations, highlighting a gap in research regarding other demographic groups.

While the use of patient portals has increased over the years, it remains most common among individuals with high literacy, high education levels, and those who are White; for instance, an analysis of the 2011-2015 National Health Interview Survey showed a 36% increase in the use of eHealth services (eg, web-based medical appointments, prescription refills, and email communication with health care providers) among immigrants compared to an increase of 53% among individuals born in the United States [8]. Notably, immigrants with LEP were 81% less likely to use these eHealth services (adjusted odds ratio 0.19, 95% CI 0.09-0.42) than those who spoke English proficiently [8].

### Objectives

Despite these disparities, little is known about how individuals with LEP perceive health IT, particularly patient portals, in terms of relevance, feasibility, and impact. To address this gap, the goal of this study was to better understand immigrant women’s experiences and perceptions regarding patient portals. Immigrant women face more challenges than immigrant men in accessing respectful and culturally competent health care [9]. By focusing on some of the common languages spoken in households in the United States [10]—Korean, Spanish, and Swahili—this study aimed to (1) explore the perspectives and beliefs about patient portals among women with LEP and (2) describe potential barriers and facilitators to their use in meeting these women’s needs.

## Methods

### Design and Sample

We used a qualitative semistructured interview design to describe the experiences and perspectives of women with LEP in using patient portals for health promotion. This approach allows researchers to assess what respondents describe as aspects of their culture that influence their use of technology for health, in their own words and context, without having constrained categories [11]. This is an extremely valuable approach in cross-cultural work with ethnic minority groups, in that the interviewer is free to pursue topics in greater depth and gain better understanding of cultural values and norms concerning a given phenomenon [12].

Participants were eligible if they (1) self-identified as women with LEP (ie, difficulty using English); (2) spoke Korean, Spanish, or Swahili; and (3) were aged ≥18 years. Participants were excluded if they had an acute or terminal condition (eg, life expectancy of <6 mo or cancer treatment within the past 5 y) or if they had either a psychiatric diagnosis (eg, schizophrenia or cognitive impairment) or other conditions that precluded participation in study activities. The study team reached data saturation after interviewing 12 (43%), 9 (32%), and 7 (25%) Korean-, Spanish-, and Swahili-speaking women, respectively, yielding a total of 28 women with LEP in the study.

### Procedures

Given that the study sample involved diverse linguistic and cultural groups of women with LEP, the study team included trained bilingual research staff for each language group, who recruited and interviewed eligible women in their respective languages. Specifically, the research staff distributed study flyers in the 3 target languages through mobile messaging apps, such as WhatsApp (Meta) for Swahili-speaking women, Kakao Talk (Kakao Corporation) for Korean-speaking women, and ResearchMatch (a web-based national registry to connect volunteers to study teams [13]) for Spanish-speaking women, as well as ethnic churches. We also used word of mouth to recruit participants. Once potential participants were identified, bilingual staff sent them a Qualtrics (Qualtrics International Inc) survey link through email or messaging apps and asked them to complete the survey. The Qualtrics survey verified eligibility for participation, and for those who confirmed eligibility, the link provided pertinent consent information followed by a brief survey addressing sociodemographic characteristics and prior use of patient portals. The brief survey also allowed women to select tentative days and times for the study interview, which was followed up by bilingual staff.

Interviews were conducted via Zoom (Zoom Video Communications, Inc) or in person, based on participant preference. All interviews were conducted in the participant’s native language. We developed a semistructured interview guide that covered topics including past experiences with using patient portals for those with prior patient portal use, perceived feasibility and relevance of patient portals among those without prior patient portal use, and perceptions of patient portals targeted toward immigrant women with LEP for health promotion. Sample questions for individuals with prior use of the patient portal included “Tell me about your experience with the patient portal” and “What/who helped you decide to use the patient portal?” Participants were presented with screenshots of patient portal features throughout the interview to facilitate the discussion. Individuals without prior patient portal use were first presented with screenshots of sample patient portal features and informed about its current functionalities. Sample questions for nonusers included “How useful do you think a patient portal would be to meet your needs?” “What device would you likely use to access the patient portal?” (refer to Textbox 1 for example interview questions). Interview guides were originally developed in English and translated into the 3 target languages by the research staff. Each interview lasted 30 to 45 minutes and was audio recorded to facilitate verbatim transcription and analysis. Each bilingual interviewer reached data saturation after interviewing 12 (43%), 9 (32%), and 7 (25%) Korean-, Spanish-, and Swahili-speaking women, respectively, among the 28 participants.

**Example interview questions.**

**For women with prior patient portal use**

- Tell me about your experience with the patient portal.
  How was it introduced? Has your provider discussed your health conditions or results via patient portal?
  Was it available in your language? How did you feel about it?
- Tell me about what you gained from using the patient portal.
  What are some benefits of having health technology like patient portals?
- What did you like least about the patient portal?
  In making the patient portal better or more useful for you, what should be added, changed, or removed from it?

**For women without prior patient portal use**

- What would help you decide to use or to not use a patient portal?
  What or who would help you feel comfortable using it?
- What do you think about the patient portal?
  How useful do you think a patient portal would be to meet your needs (eg, looking up medication, finding out diagnosis, obtaining laboratory results, and cancer screening)?
  How would you want to be informed or educated on how to use a patient portal?

### Ethical Considerations

The study was approved by the Johns Hopkins Medicine Institutional Review Board (IRB00243015). Verbal informed consent was obtained from all study participants before data collection, either over the telephone or in person. The consent included information on the risks and benefits of participating in the study. Every potential participant was given a chance to ask questions about their involvement in the study before consenting. To maintain participants’ confidentiality, the audio-recorded interviews were exported to a secure cloud storage service (Johns Hopkins OneDrive) immediately after the interviews and deleted from the original recording device. Upon interview completion, participants were sent a US $30 gift code for their participation.

### Analysis

We used descriptive statistics to summarize sample characteristics collected from a brief sociodemographic survey*.* The findings from the qualitative interviews were used to identify descriptors and expressions related to the patient portal experiences of women with LEP. The first author and the coders met regularly to discuss the themes and subthemes and resolve any discrepancies.

The bilingual data collectors transcribed the audio-recorded interviews into Microsoft Word documents and individually coded the data in the original languages to minimize the loss of participants’ intended meanings of words, phrases, and concepts [14]. We used a deductive coding based on a priori topics used to develop the interview guide and applied an inductive coding approach, allowing the codes to emerge from the data during the coding process [15]. The coders met regularly to discuss the themes and subthemes that emerged from the data. Data saturation was determined when no new themes emerged from the analysis. The findings from the qualitative interviews were used to identify common themes and subthemes among prior users and nonusers of patient portals. Finally, all themes, subthemes, and quotes were translated into English and merged in a Microsoft Excel spreadsheet.

## Results

### Sample Characteristics

Table 1 presents the characteristics of the interview participants. Half (14/28, 50%) of the participants were in their 30s and 40s, with the average ages being 37 (SD 12), 42 (SD 11), and 48 (SD 15) years for Korean-, Spanish-, and Swahili-speaking women, respectively. Korean-speaking participants had the longest average length of stay in the United States (15, SD 11 y), followed by Spanish- (12, SD 9 y) and Swahili-speaking (5, SD 2 y) women. All Spanish-speaking participants (9/9, 100%) and most of the Korean-speaking women (11/12, 92%) had received a high school or college education, whereas more than half of the Swahili-speaking participants (4/7, 57%) had only an elementary-level education. All Korean- and Swahili-speaking women identified themselves as non-Hispanic Asian and African American, respectively, whereas 89% (8/9) of the Spanish-speaking women identified themselves as Hispanic. The interview participants originated from Central America (Dominican Republic, Colombia, and Venezuela), South Korea, and sub-Saharan Africa (Democratic Republic of the Congo and Kenya). Prior use of patient portals varied: 58% (7/12) and 44% (4/9) of the Korean- and Spanish-speaking participants, respectively, had prior experience, whereas all Swahili participants (7/7, 100%) had no prior exposure to patient portals.

**Table 1:** Sample characteristics (N=28).

Characteristics		Korean-speaking women (n=12)	Spanish-speaking women (n=9)	Swahili-speaking women (n=7)
Age (y), mean (SD; range)		37 (12; 23-61)	42 (11; 23-56)	48 (15; 27-70)
Length of stay in the United States (y), mean (SD; range)		15 (11; 1-36)	12 (9; 1-30)	5 (2; 2-6)
**Education, n (%)**				
	Elementary school	0 (0)	0 (0)	4 (57)
	Middle school	0 (0)	0 (0)	1 (14)
	High school	1 (8)	5 (56)	1 (14)
	College	10 (83)	4 (44)	1 (14)
	Graduate school	1 (8)	0 (0)	0 (0)
**Race and ethnicity, n (%)**				
	African	0 (0)	1 (11)	7 (100)
	Asian	12 (100)	0 (0)	0 (0)
	Hispanic	0 (0)	8 (89)	0 (0)
**Country of birth, n (%)**				
	Democratic Republic of the Congo	0 (0)	0 (0)	4 (57)
	Dominican Republic	0 (0)	3 (33)	0 (0)
	Colombia	0 (0)	1 (11)	0 (0)
	Kenya	0 (0)	0 (0)	3 (43)
	South Korea	12 (100)	0 (0)	0 (0)
	Venezuela	0 (0)	4 (44)	0 (0)
	No response	0 (0)	1 (11)	0 (0)
Prior patient portal use, n (%)		7 (58)	4 (44)	0 (0)

### Themes and Subthemes

#### Overview

Multimedia Appendix 1 summarizes the findings on perceptions and experiences with patient portal use, preferred features, and suggested improvements, organized by themes and subthemes with illustrative quotes. We identified 4 main themes that were common across the linguistic groups: perceived benefits of patient portals, perceived facilitators of patient portal use, perceived barriers to patient portal use, and preferred features and suggested improvements. While most subthemes were shared across all 3 groups, some were unique to specific groups, as detailed in the subsections that follow.

#### Theme 1: Perceived Benefits of Patient Portals

Participants acknowledged and described multiple benefits of using patient portals. Perceived benefits of patient portals included easier communication with health care providers and health systems, getting connected and staying connected with health systems, easier and efficient access to one’s health records over time, staying informed of and engaged with one’s health and health management, and better patient engagement in medical visits.

##### Easier Communication With Health Care Providers and Health Systems

Patient portals were perceived as effective and efficient tools for facilitating communication with the health system. Both users and nonusers noted that these portals increase efficiency and reduce unnecessary in-person visits to health care providers. A Swahili-speaking participant in her late 20s perceived that patient portals might be helpful tools for communicating with health care providers, accessing health information, and ultimately reducing the need for frequent in-person hospital visits:

It may help you communicate with the health care provider...it makes everything easier; you don’t have to frequently go to the hospital to get information concerning your health from the health care provider.

SIDSW05

A Korean-speaking participant in her late 40s noted that patient portals enable women with LEP to take as much time as needed to understand health information, including searching for the meanings of terms using translation tools. She pointed out that some terms can easily be missed or misunderstood during conversations:

[I]t’s better to use the patient portal so that we [people with LEP] can take our own time to dig into the information. We need more time to understand the information because it’s challenging to grasp everything during a conversation. By using the patient portal, we can search for terms and use translation tools. I think it’s better to use it rather than feeling pressured to understand everything all at once.

SIDKO01

##### Getting Connected and Staying Connected With Health Systems

The health system felt distant to many participants not only due to various barriers but also because of the difficulty in becoming familiar with a health system different from the one they had experienced in their home countries. Many participants expressed that the patient portal made staying connected with the health system and accessing check-ups a smoother process. A Spanish-speaking participant noted that she visits the patient portal at least once a month to schedule or monitor upcoming appointments:

At least once a month I would check if I had any upcoming appointments, if I need to schedule a new appointment, if my annual exam is approaching, or if I need to see my gynecologist.

SIDSP05

Similarly, a highly educated Korean woman in her early 50s recalled feeling hesitant when she first encountered the patient portal. However, over time, she gained familiarity and appreciation for it. She stated as follows:

As I kept using [the patient portal], it became easier to use and...then I realized it doesn’t take that much time to get the check-ups [scheduled]...the scope of my medical examination has expanded.

SIDKO02

##### Easier and Efficient Access to One’s Health Records Over Time

One of the main benefits highlighted by the participants was that patient portals serve as a convenient way to keep their health records in one place, allowing easy access to their information. Patient portal users shared their positive experiences, contrasting them with past struggles in tracking health information such as immunization records, laboratory results, or medication history. A Korean-speaking participant in her 40s noted that she finds it convenient to have all her health records in one place, readily available whenever she needs to check her test results:

It’s good that my medical history or information is stored in one place. I can go back to my records and check when I received certain tests.

SIDKO01

Efficiency and ease of information access was echoed by a Spanish-speaking participant:

A great benefit of patient portals is that I have access to my information immediately...I think the patient portal is very accessible because it lets me see all of my test results.

SIDSP05

##### Staying Informed of and Engaged With One’s Health and Health Management

This subtheme, focusing on managing one’s health records in one place, contributed to greater awareness of one’s health. Having easy access to their records allowed individuals to become knowledgeable about their health status, motivating them to take a more active role in managing their health. As noted by a Spanish-speaking participant, the patient portal gave patients the autonomy to access and stay informed about their records at their convenience:

Accessing my medical records [through the patient portal] gives me a lot of independence. I can use [the portal] whenever I want or whenever I can, knowing that I will always have access.

SIDSP08

Parallel to this benefit, a Korean-speaking participant emphasized that the patient portal is a valuable tool for looking up one’s test results and proactively managing one’s health. She stated as follows:

I think it’s easier to stay informed and reminded of my health status when I can see my own [test] results. By looking at my results, I gain more awareness and can manage my health better.

SIDKO03

In addition, a Swahili-speaking participant highlighted the benefits of patient portal access in improving engagement in care:

[I]t would be beneficial in communicating with the health care provider; understanding how to get medication, and how to follow up with the health care provider.

SIDSW04

##### Better Patient Engagement in Medical Visits

Participants viewed the patient portal as a valuable tool for accessing health information before and after medical visits. They described experiencing a higher level of stress when communicating in English about vital health matters compared to their experiences in their home countries. The patient portal allowed them to anticipate discussions, prepare questions, and review results at their convenience. A Korean-speaking participant in her 30s mentioned that reviewing her results before her appointment helped facilitate a productive discussion with her health care provider:

I can have a better understanding before the appointment. If I go to see the doctor without knowing anything, there might be parts I don’t understand. However, if I already know the results in advance, it would be easier for me to comprehend and discuss with the doctor during the appointment.

SIDKO10

Along the same lines, Spanish- and Swahili-speaking participants viewed patient portals as a valuable digital tool for following up with health care providers. A Spanish-speaking participant noted that the patient portal is useful for seeking clarification after appointments:

There are times during your appointment that you forget to ask [questions], so having the patient portal and being able to go back and check it whenever is a great advantage.

SIDSP06

#### Theme 2: Perceived Facilitators of Patient Portal Use

Participants identified various factors that facilitated their continued use of patient portals, rather than initial adoption. These included availability of time, widespread use and availability of smartphones and the internet in the United States, family support, and parenthood.

##### Availability of Time

Spanish- and Swahili-speaking participants affirmed that availability of time played a role in facilitating the use of patient portals. Some Swahili-speaking participants also noted that availability of time may be a good motivator for learning how to use patient portals as a source of health information and for seeking care. No relevant quotes were identified for Korean-speaking participants. A Spanish-speaking participant mentioned that availability of time increased the likelihood of using patient portals to view results or schedule appointments:

Having the time to make appointments and access your tests results...is a facilitator of patient portal use.

SIDSP03

##### Widespread Use and Availability of Smartphones and the Internet in the United States

Of note, Swahili-speaking participants perceived that the extensive use and availability of smartphones and the internet in the United States enhanced patient portal use. This subtheme was unique to the Swahili-speaking group. A Swahili-speaking participant stated as follows:

There is no one in America who does not have a phone, I was even given a computer when I immigrated here, it’s just that I don’t know how to use it.

SIDSW07

##### Family Support

Participants perceived that support from family members would facilitate their access to and use of patient portals. A Korean-speaking participant disclosed that she would seek help from her son on how to use the patient portal and was confident that she would know how to use it after a few trials:

I’ll ask my son whenever there’s something I don’t know, and then once I practice a couple of times, I can use it on my own.

SIDKO07

Notably, most of the Korean-speaking participants in their 20s to 40s expressed concerns that their parents would face difficulties accessing the patient portal. Consequently, they expected to provide support to their parents. Similarly, a Swahili-speaking participant who was illiterate shared that either her children or spouse would help her use the patient portal if needed. Consistent with these perceptions, a Spanish-speaking participant in her 50s (a nonuser) reported that she would like a family member to show her how to use a patient portal:

It would be more comfortable if someone in my home taught me [how to use the patient portal], like a family member.

SIDSP03

##### Parenthood

Having school-age children acted as a facilitator for patient portal use among Korean- and Spanish-speaking participants with prior portal experience. No relevant quotes were identified for Swahili-speaking participants or for those without prior patient portal use. All 3 Korean-speaking participants who had school-age children emphasized that convenient access to their children’s health care records was a key facilitator. While recent immigration could have been a barrier, parenthood seemed to help them quickly adapt to the US culture and health system:

With the patient portal, it seems to be an efficient way to connect with their [children’s] health care providers, especially for simple questions and check-ups. That’s my first impression of it.

SIDKO1

Similarly, a Spanish-speaking participant mentioned that, despite difficulties communicating with staff at the physician’s office, she was able to schedule her son’s medical appointment using the patient portal:

Not having the ability to speak English has made everything more difficult. For example, I needed to schedule an appointment for my son to see the doctor. When I called, everyone spoke English, and I didn’t understand anything. So, I used my patient portal to open his account, and through there I was able to accomplish it [schedule her son’s appointment].

SIDSP09

#### Theme 3: Perceived Barriers to Patient Portal Use

Participants mentioned several barriers to using the patient portal, including limited digital literacy and limited access to technology; LEP; lack of awareness and knowledge about patient portals; and, additionally, for Swahili-speaking participants, illiteracy.

##### Limited Digital Literacy and Limited Access to Technology

Digital illiteracy, often stemming from limited access to digital devices and the internet, was mentioned as a potential navigational challenge for interested users. Many participants expressed that a lack of knowledge on how to navigate the patient portal would be a key barrier to its use; for example, some Korean-speaking participants shared that their older adult parents or relatives would not use the patient portal:

When I think about my aunt, she’s not familiar with using smartphones or websites at all. First of all, she forgets her username and password. She dislikes the process of retrieving passwords...Navigating through websites is also difficult for her, whether it’s in English or Korean, because there are too many menus. Our portal also has menus, but when you click one, several options pop up. Also, the fonts are small.

SIDKO12

##### LEP Issue

LEP was identified as a profound barrier to patient portal access and use. Women with prior patient portal experience noted that the lack of patient portal access through a language-concordant medical practitioner is a major barrier for many women with LEP. For some, LEP was an even greater challenge than limited digital literacy. A Korean-speaking participant suggested that LEP can undermine self-efficacy in using the patient portal, even among those who are knowledgeable about it and have access to digital devices:

If English proficiency is limited, even with well-functioning devices, websites, or apps that are used in English, there is still some fear and uncertainty because of the fixed idea that ‘I don’t know’ or ‘I don’t understand.’ Even if there is knowledge of the existence of such portals, it’s still intimidating to try because of the discomfort with English.

SIDKO05

Similarly, a Spanish-speaking participant observed that while the patient portal may be accessible in Spanish, the presentation of results in English limits its usability:

Honestly, I think the portal is really good because at least it gives you the option to put it in Spanish. That way, it isn’t as hard to follow the steps to create your account. However, your tests results are always in English, which always leaves me thinking ‘how do I read these?’ Even when I try to translate the results, it is not the same [as having it in Spanish]...The doctor could call you and tell you ‘Everything is okay,’ but that is not enough.

SIDSP09

Patient portals are not yet available in other ethnic languages, such as Swahili, which hinders access and use. This concern was highlighted by a Swahili-speaking participant who had no prior experience using a patient portal**:**

Patient portal would be more useful if it is written in both English and Swahili, since not everyone understands English.

SIDSW05

##### Lack of Awareness and Knowledge About Patient Portals

Participants noted that, despite the multiple benefits highlighted, patient portals are generally not well known within their communities*.* They shared that increasing awareness would likely encourage interest and use. A few Korean-speaking participants emphasized that greater awareness could drive adoption:

As long as one knows about the patient portal, it’s something that would be definitely utilized. But I didn’t know...Since I now know, I’m going to use it.

SIDKO06

##### Illiteracy

Illiteracy was identified as a key barrier to patient portal use among Swahili-speaking participants. No other group noted this as a barrier. A Swahili-speaking participant expressed interest in using the portal but cited limited reading ability as a significant obstacle:

If patient portal is written in Swahili, I wouldn’t know how to read well, I just know a few words, therefore, having information in audio or video formats would be useful since I don’t know how to read.

SIDSW06

#### Theme 4: Preferred Features and Suggested Improvements

Participants also shared suggestions to enhance the patient portal user experience, focusing on language accommodation, device access, features, formatting, and specific health information preferences.

##### Expanded Language Access to Accommodate Non-English Speakers

Most participants emphasized the need for expanded language access to facilitate patient portal use. Across all language groups, they expressed a preference for patient portals available in their preferred languages. A Spanish-speaking participant noted that since LEP is a major barrier to patient portal use, offering access in preferred languages would be ideal:

Not knowing the language [English] is the main reason why people can’t use the patient portal. Ideally, the portal should be able to be used in different languages depending on the person’s preference.

SIDSP08

Her sentiments mirrored those of a Korean-speaking participant:

I hope these features can be available in Korean. I wish for patient portals to be accessible in Korean so that even those who struggle with new technologies or have difficulty as they age can still have the opportunity to try it in their native language.

SIDKO07

In this context, another participant discussed the issue of understanding medical terminology (ie, health literacy) and noted that it would be helpful if information was provided in Korean because medical terms are more difficult to comprehend in English:

Since I’m not familiar with medical terms, if something is written only in English, I wouldn’t understand. It would be helpful to have more detailed explanations.

SIDKO09

In addition, a Swahili-speaking participant affirmed the need for expanded language access:

The patient portal will be more useful if it is written in Swahili.

SIDSW06

##### Improved Accessibility to Health Information Using Graphics and Patient Education Materials

Several participants wished for features that would enhance understanding, such as comparisons of laboratory test results, explanations of medications (eg, precautions and contraindications), and detailed interpretations of laboratory results using graphs or charts. A Korean-speaking participant asserted that displaying laboratory values in graphs or charts would be beneficial:

It would be much more convenient if there were a chart or graph showing last year’s lab values compared to this year’s values. For example, if there were red arrows indicating how much they went up or down compared to last year, that would be really helpful.

SIDKO05

Likewise, a Spanish-speaking participant stated that the patient portal should be able to provide basic, patient-centered information about health status:

[A]t least check my health status. To see if I am allergic to something; if I have any upcoming appointments; if I am taking any medication, and if so what kind, how much of it, and how many times a day...Basic things like that.

SIDSP05

In addition, Swahili-speaking participants expressed a desire for the inclusion of pictures and audio-recorded files to improve accessibility to health information on the patient portal:

[P]ictures and audios, with texts written in simple English or even Swahili.

SIDSW03

##### User Onboarding Education and Technical Support

Participants’ perceived needs for using the patient portal were similar; many suggested that user onboarding education and technical support are necessary improvements for broader adoption. Regarding the format of onboarding education, some participants expressed a preference for audiovisual tools, while others were comfortable receiving links to instructional content. A Korean-speaking participant mentioned that an internet-based demonstration would be sufficient for her understanding:

I think [getting instructions] via email and a link to view the video about it would be helpful.

SIDKO09

Considering limitations in technology access and use as well as English proficiency, participants noted that printed instructions in their native language or in-person guidance would be helpful for older adults:

I would like to be instructed by someone, for instance, after church service.

SIDSW06

Finally, recognizing the constraints of physicians’ busy schedules, a Korean-speaking participant stated that a brief demonstration during an appointment would be highly beneficial:

It’s best if the doctor explains how to use the patient portal, but they’re busy. If they open my portal during my visit and let me know ‘You can see it on this portal,’ I’d be more motivated to use it.

SIDKO05

## Discussion

### Principal Findings

Despite the rapid adoption of patient portals in health care settings [7,8], research on the experiences and perspectives of individuals with LEP is scarce. Our sample of women with LEP from 3 cultural and linguistic groups provided valuable insights into their perceptions, experiences, and beliefs regarding patient portals. The findings highlight the benefits, facilitators, and barriers to using patient portals, as well as preferences for specific features and suggestions for improvements.

Participants with LEP strongly associated patient portals with various perceived benefits, including easier communication with health systems and health care providers and better engagement in medical visits. These perceived benefits are similar to those reported in a recent analysis of comments by predominantly non-Hispanic White female patients in their 50s who had used a patient portal in a health system [16]. According to the analysis, most patients highlighted communicating with health care teams and monitoring health status and care activities as beneficial functions of patient portal use. Especially for women with LEP, the ability to review their records and take as much time as needed to understand health information, including searching for the meanings of terms using translation tools, were considered an important benefit of patient portals. Without this option, understanding health information during medical conversations would be challenging. Patient portals are generally considered beneficial to improving patient engagement in health management [17-19]. As patient engagement is increasingly recognized as an essential component of person-centered care [18], our findings suggest that providing women with LEP access to a patient portal could be a viable approach to promote their engagement in health management and care, potentially leading to positive health outcomes [18].

While the subthemes for facilitators of patient portal use (eg, availability of time, widespread use and availability of smartphones and the internet in the United States, family support, and parenthood) varied across groups, family support emerged as a common subtheme across all 3 groups. A cohort study conducted to assess the prevalence of health care portal use before, during, and after the COVID-19 pandemic and involving middle-aged and older adult primary care patients (2019-2022) [20] found that portal log-in activity was higher during the pandemic compared to the 2019 baseline: higher portal log-in activity was associated with adequate health literacy and multimorbidity, whereas lower portal log-in activity was associated with older age and female sex. Disparities associated with age and sex decreased as the pandemic progressed, but disparities based on health literacy were exacerbated. The subtheme of family support identified in our study highlights how women with LEP navigate limited health literacy related to patient portals by mobilizing resources within their reach, such as children. Evidence points to the unique role of children in immigrant families as language brokers who “translate and mediate between their heritage language/culture and English/US culture” for their parents with LEP [21]. A similar pattern has been reported in the context of digital health where children help their immigrant parents to access web-based health resources [22]. Nevertheless, research in this area is scarce and has primarily relied on qualitative study designs. Future research is warranted to investigate how health literacy develops and is applied to digital health access in immigrant families, as well as the role bilingual children play in obtaining, processing, and understanding web-based health information to support family members, such as parents with LEP.

While patient portals enable women with LEP to communicate effectively with health care providers and health systems, several barriers identified in this study were closely linked to social determinants of health, including limited digital literacy and limited access to technology, LEP, and illiteracy. Similar barriers to patient portal use have been reported; for example, a recent cross-sectional study of 1850 respondents in the United States found that digital health literacy, home internet access, access to technology devices, and English proficiency were among the correlates of patient portal enrollment and intent to use [23]. Similarly, another study using data from the 2017-2018 Health Information National Trends Survey revealed that LEP, along with no health insurance, limited education, and not having a regular doctor, was significantly associated with lower patient portal use [24]. Of note, several reviews reported concerns about privacy and data security as major barriers to patient portal use. However, these concerns did not emerge as subthemes among the women with LEP in this study. One possible explanation is that our study sample included both women with prior patient portal use and those without, whereas previous research often involved patients already engaged with a health system. It would be important to consider how attitudes and perceptions of patient portals in ethical domains (eg, privacy concerns and data security) might hinder or facilitate patient portal adoption and meaningful use among populations with LEP [25].

Key improvements to patient portals suggested by the women with LEP in this study included expanded language access to accommodate non-English speakers, improved accessibility to health information using graphics and patient education materials, and user onboarding education and technical support. An innovative patient portal that transcends critical barriers such as LEP can be an empowering tool, especially for immigrant populations, to obtain health information, manage care, increase knowledge, and take health care ownership. In addition, the suggestions for the use of graphics and educational materials as well as user onboarding education and technical support underscore the need for improved usability, making patient portals more accessible and user-friendly. This also highlights the importance of more active and direct engagement and endorsement by the health system and health care providers [26,27]. Interactions with patient portals have been widely studied; yet, the influence of usability on patients’ decision-making remains understudied [28]. Future research should take a comprehensive approach to examining usability and its effect on patient portal use among the growing and increasingly diverse patient population with LEP.

### Limitations

This study has some limitations. First, as a qualitative study, the interpretations are inherently subjective, and it is also possible that social desirability bias during the interviews influenced participants’ responses. Second, we did not examine participants’ type of insurance, which could be associated with immigration status and may present additional barriers to equitable health care access. Insurance coverage seems to play a role in determining whether participants use patient portals [24]. Third, English proficiency was self-reported and may be subject to self-report bias. In addition, our study sample primarily consisted of younger individuals with more than a decade of residence in the United States and high levels of education, except for the Swahili-speaking group, necessitating careful interpretation of the findings. Their experiences were also specific to the US health care context. In addition, part of the recruitment process relied on technology such as WhatsApp, websites, and Zoom, which may have excluded individuals without such access. Future research should strive to include a more diverse sample to capture a broader range of perspectives and experiences. Finally, the study included women without prior patient portal use, primarily Swahili-speaking participants. The interpretability of the findings, particularly among those without prior exposure to patient portals, may be limited.

### Conclusions

Women with LEP acknowledged several benefits of patient portals but also identified barriers to their use, including limited digital literacy, restricted access to technology, LEP, and illiteracy. These barriers are closely linked to social determinants of health, which disproportionately affect women with LEP. As digital health continues to evolve, it is critical for health systems to address digital health disparities and foster meaningful use. This study is novel in its inclusion of women with LEP, providing unique insights into their perceptions, beliefs, and experiences with patient portals. One of the Healthy People 2030 goals is to “eliminate health disparities, achieve health equity, and attain health literacy to improve the health and well-being of all” [29]. Our findings suggest that this goal may be supported by promoting accessible health resources, such as patient portals, with several considerations to enhance usability for populations with LEP. Emerging evidence highlights the significance of presentation formats in improving patient satisfaction and usability [30], and our findings could inform future research to promote engagement with patient portals among individuals with LEP.

## Appendix

Multimedia Appendix 1 Themes, subthemes, and illustrative translated quotes for each group.

## Abbreviations

LEP: limited English proficiency

## Data Availability

Interview data in local languages are available from the corresponding author on reasonable request.
