# Supplementary material for: Perceptions, Experiences, and Beliefs About Patient Portals Among Women With Limited English Proficiency: Multicultural Qualitative Interview Study
Source: J Med Internet Res. 2025 Feb 26;27:e60699. doi: 10.2196/60699 (PMC11904362; doi:10.2196/60699)
Supplement: Multimedia Appendix 1 [file jmir_v27i1e60699_app1.docx]

**Multimedia Appendix 1.** Themes, subthemes, and illustrative translated quotes for each group.

| Theme and subtheme | Korean-speaking women | Spanish-speaking women | Swahili-speaking women |
| --- | --- | --- | --- |
| **Theme 1: perceived benefits of patient portals** | | | |
| Easier communication with health care providers and health systems | - “The biggest advantage is that it’s not hard to conveniently book and cancel appointments.” [SIDKO06] | - “Patient portals save you a lot of time. Often when you call [the physician’s office], they make you wait a while. The process [of calling] is tedious because first you must speak with the receptionist. Then if someone else is in line, you must wait for your turn.” [SIDSP03] | - “Patient portals will help me communicate with the doctor... Sometimes the doctor’s appointment schedule is full, and it takes months for your appointment date.” [SIDSW01] - “It would help me communicate with the health care provider and to see my test results, but I would still need to get explanations of those results.” [SIDSW07] |
| Getting connected and staying connected with health systems | - “When I got a blood test, the results showed numerical values and whether they were within the normal range or not, and whether it was negative or positive. So, it was a good experience because I could see everything at a glance.” [SIDKO09] | - “[Patient portals] would inform you more thoroughly about things. For example, if you have to do a mammogram, [the portal] will provide you with detailed information like what test they are going to perform on you...and why do you need it.” [SIDSP02] | - “I see that [patient portals] are good because you will find information there and you might learn something you didn’t know.” [SIDSW02] |
| Easier and efficient access to one’s health records over time | - “I personally love being able to track these test results and keep looking at them for years. I also love being able to see if I missed any appointments or check when the last time was, I visited.” [SIDKO05] | - “Of course, it would be easier [to have a patient portal over going to the physician’s office in person]. You just have to open your web browser and all your information is there, instead of on a piece of paper.” [SIDSP06] | - “If I had a patient portal, I would know my health status... If the patient portal is on my phone, I can access it anytime, without the need to travel far [to my health care provider].” [SIDSW03] |
| Staying informed of and engaged with one’s health and health management | - “The patient portal served as motivation for my health. It gave me a certain level of awareness about my well-being.” [SIDKO07] - “When I see improvements in my HbA_1C_ levels, it motivates me to put more effort into it. I remember being thrilled when my anemia returned to normal levels. It was like, ‘Wow!’ I was so happy.” [SIDKO12] | - “From what I could see, the portal contains all of the aspects of your health information, and it can tell you if your health status is good, bad, or intermediate. I imagine it also shows your weight, or if you have to improve something [in your health]. I think it’s [something] very fruitful for the person.” [SIDSP05] | - “[If you have a patient portal] you will look for information about diseases, signs and symptoms of diseases, how to prevent diseases.” [SIDSW01] |
| Better patient engagement in medical visits | - “I prefer to have a grasp and understanding through the patient portal before meeting with the doctor. At first, I can review and understand the information, and then when the doctor explains, I can have a more constructive conversation and ask more questions.” [SIDKO11] | - “The information [on the patient portal] is more complete. If it is well-written, it is better to have [your information] online because one can go back to the portal at any time if you have doubts or questions.” [SIDSP06] | - “Patient portals are good because after seeing the health care provider you can follow up for things that don’t need an in-person visit.” [SIDSW05] |
| **Theme 2: perceived facilitators of patient portal use** | | | |
| Availability of time | - NR^a^ | - “I would be interested in learning how to use the patient portal because I have the time.” [SIDSP05] | - “If you are working so many days [in a week], you will not have time to read information about your health [on the patient portal].” [SIDSW07] |
| Widespread use and availability of smartphones and the internet in the United States | - NR | - NR | - “Here in America, the internet is always there, [accessing the patient portal] might only be a problem in the countries where we are originally from.” [SIDSW02] |
| Family support | - “I think that if it [the patient portal] is not available in our native language, then our parents’ generation might find it [using the patient portal] a bit difficult.” [SIDKO10] | - “My kids [would help me].” [SIDSP02] | - “[If I need help using the patient portal] the lady I work for, or my niece, would help me.” [SIDSW01] |
| Parenthood | - “My daughter has some severe allergies and skin issues and goes to a dermatologist who uses the same patient portal as mine. It was convenient for me because I could easily access and navigate to see all her health information, all in one place.” [SIDKO1] | - “I opened the patient portal with my husband. Together, we created mine, his, and my son’s accounts.” [SIDSP09] | - NR |
| **Theme 3: perceived barriers to patient portal use** | | | |
| Limited digital literacy and limited access to technology | - “As people age, they tend to lose interest in learning new technologies and prefer simpler lives, focusing on other aspects such as financial matters and retirement.” [SIDKO07] | - “The problem is that not everyone knows how to work with computers and navigate the web. Many times people want to, but if they don’t understand, they won’t be able to access [the patient portal].” [SIDSP02] | - “Not knowing how to use [the patient portal]; open here, click there, how to log out, how it works, how to read it [would prevent women with limited English proficiency from using the patient portal].” [SIDSW01] |
| Limited English proficiency | - “If the information is presented solely in English, I might not fully comprehend it, leading to heightened anxiety.” [SIDKO06] | - “What really limits us is the language. That’s what makes it complicated. It’s happened to me where I download an app and because it is in English, I stop using it.” [SIDSP05] | - “Not being proficient in English [would hinder immigrant women from using the patient portal].” [SIDSW07] |
| Lack of awareness and knowledge about patient portals | - “I hope more people use the portal to minimize the potential of misunderstanding their health information.” [SIDKO04] | - NR | - “If they [immigrant women with limited English proficiency] don’t know about the patient portal [it would prevent them from using it]. So many people want to be educated. As you can see, I didn’t know about the patient portal.” [SIDSW03] |
| Illiteracy | - NR | - NR | - “Not knowing how to read [would prevent immigrant women with limited English proficiency from using the patient portal].” [SIDSW02] |
| **Theme 4: preferred features and suggested improvements** | | | |
| Expanded language access to accommodate non-English speakers | - “I hope the medication names are not all in English; it would be better to have them in parentheses with the Korean names as well. It would be more convenient to access and understand faster.” [SIDKO12] | - “I think what they are doing is excellent, truly. The fact that everyone can have access to the patient portal is spectacular... but many of us have a lot of trouble using the portal. It would be much better if it was all in Spanish so we could all have the opportunity to use it.” [SIDSP07] | - “[It would be nice to have] pictures and audios, with texts written in simple English or even Swahili.” [SIDSW03] |
| Improved accessibility to health information using graphics and patient education materials | - “It would be really helpful if there were explanations in the portal about what precautions to take, potential kidney issues, which medications shouldn’t be taken together, and when to take the medications.” [SIDKO05] | - “If the information is very detailed, meaning the medical information is a lot, it would be nice to have multiple tabs. But if it is not a lot of information, one window is fine.” [SIDSP06] | - “I would like for the patient portal to have pictures that I can see.” [SIDSW02] |
| User onboarding education and technical support | - “If there is a guideline booklet like that in the beginning, as a printout in Korean, I think it would be nice.” [SIDKO05] - “It’d be helpful if there are short clips or instructions [on how to use the patient portal].” [SIDKO12] | - “Maybe online like how we are speaking right now [Zoom]. It would be nice if they showed me how to use the portal on the screen.” [SIDSP06] | - “[H]aving a link is okay, a video is also okay, but I prefer a link.” [SIDSW04] - “If you send me an audio recording [on how to use the patient portal].” [SIDSW06] |

^a^NR: not reported.
